# Characteristics of Articles About Human Papillomavirus Vaccination in Japanese Newspapers: Time-Series Analysis Study

**DOI:** 10.2196/publichealth.8237

**Published:** 2017-12-19

**Authors:** Nao Ueda, Ryoki Yokouchi, Taro Onoda, Atsushi Ogihara

**Affiliations:** ^1^ Graduate School of Human Sciences Waseda University Tokorozawa Japan; ^2^ Department of Health Sciences and Social Welfare School of Human Sciences Waseda University Tokorozawa Japan; ^3^ Department of Medicine School of Medicine Tokai University Isehara Japan; ^4^ Faculty of Human Sciences Waseda University Tokorozawwa Japan

**Keywords:** papillomavirus vaccines, immunization programs, uterine cervical neoplasms, newspapers as topic, mass media, data mining, Japan

## Abstract

**Background:**

Media coverage and reports have a major influence on individual vaccination and other health-related activities. People use the media to seek information and knowledge on health-related behaviors. They obtain health-related information from media such as television and newspapers, and they trust such information. While several studies have examined the relation between media coverage and individual health, there is a lack of studies that have analyzed media reports of health information. In particular, we have found no analyses related to cervical cancer (human papillomavirus [HPV]) vaccine.

**Objective:**

This study aimed to identify mentions of cervical cancer vaccine in Japan’s printed news media and to determine their characteristics.

**Methods:**

We used the archival databases of 2 Japanese newspapers, *Yomiuri Shimbun* (Yomidasu Rekishikan) and *Asahi Shimbun* (Kikuzo II Visual), for text mining. First, we created a database by extracting articles published between January 1, 2007, and December 31, 2014, that matched the terms “cervical cancer” AND “vaccination” in a keyword search. Then, we tallied the extracted articles based on the month of publication and number of characters in order to conduct a time-series analysis.

**Results:**

We extracted a total of 219 articles. Of these, 154 (70.3%) were positive and 51 (23.3%) were negative toward HPV vaccination. Of the 51 negative articles, 4 (7.8%) were published before June 2013, when routine vaccination was temporarily discontinued due to concerns regarding side effects, and 47 (92.2%) were published since then. The negative reports commonly cited side effects, although prior to June 2013, these issues were hardly mentioned. Although foreign media reports mentioned side effects before routine vaccination was temporarily discontinued, fewer articles mentioned side effects than recommendations for vaccination. Furthermore, on June 13, 2013, the World Health Organization’s advisory body Global Advisory Committee on Vaccine Safety issued a statement regarding the safety of HPV vaccines, but hardly any articles reported this statement. Rather, several articles were published about the side effects after June 2013.

**Conclusions:**

Since we consider media coverage to be a factor affecting human health behavior, the media should extensively report on the cost of not receiving cervical cancer vaccination, global trends concerning cervical cancer vaccination, and statements released by various agencies on the subject.

## Introduction

Vaccinations play a significant role in preventing epidemics and the spread of infectious diseases, and a vaccination rate as high as possible is required to maintain the health of an entire society [1]. Japan’s vaccination program is regulated by the Vaccination Act [2]. Although it changed from a legal obligation to an obligation requiring an effort in 1994, the current recommended vaccinations include influenza, mumps, and hepatitis B [3]. When it comes to the cervical cancer (human papillomavirus [HPV]) vaccine, public funds are being used in more than 50 countries to provide vaccinations to adolescent girls [4]. In Australia, vaccination is recommended for girls aged 12 to 13 years, and the cost of providing cervical cancer vaccination is entirely covered by public funds. Japan started providing vaccination for cervical cancer in 2010 for girls in the first grade of elementary school to the final year in high school using a grant from public funds. In Japan, Gardasil and Cervarix are the cervical cancer vaccines used. They are effective in preventing infection with HPV types 16 and 18, which are the main causes of cervical cancer. In addition, Gardasil prevents types 6 and 11 HPV, which account for 90% of the cause of condyloma acuminata [5]. By April 2013, considering the reduction in the number of cervical cancer cases, the Vaccination Act stipulated that this vaccination should be mandatory rather than optional. However, the cervical cancer vaccine has become a social issue, with lawsuits being filed due to alleged side effects [6].

From the perspective of herd immunity, one of the key factors of a vaccine’s effectiveness is the rate of vaccination. Therefore, the main concern is how to increase it. In general, the factors relating to rate increase have to do with availability of public funds to support programs and the ease of access to medical facilities. Tauil et al reported that the mother’s level of education and her socioeconomic condition had an impact on the vaccination rate [7]. Tsuchiya et al found that the mother’s age and the primary care physician’s recommendation influenced the vaccination rate [8].

Media coverage and reports are also major factors in influencing individual vaccination and other health-related activities. People use the media to seek information and knowledge on health-related behaviors [9]. A study by Hagihara et al found that coverage of suicides in newspapers is significantly correlated to the number of suicides committed the following month [10]. Ishii reported that the number of suicides increased after articles were published on a celebrity’s suicide [11]. According to Uesugi, people obtain health-related information from media such as television and newspapers, and they trust such information [12]. On the other hand, Fujioka stated that people may also be skeptical or even critical of health-related activities reported in the media [13].

While several studies have examined the relation between media coverage and individual health, studies analyzing media reports of health information are lacking. In particular, there are few analyses related to cervical cancer vaccine [14,15]. Further studies are needed to examine the relation between media coverage and cervical cancer vaccine.

Therefore, this study aimed to identify reports of the cervical cancer vaccine in Japan’s printed news media and to determine their characteristics.

## Methods

With the increasing cross-ownership of print media and television networks in recent years, newspapers now report the same type of news as television. On this basis, for this research, we used Yomidasu-Rekishikan, the archival database of *Yomiuri Shimbun* (hereafter, *Yomiuri*), the newspaper with the largest circulation in Japan [16], and Kikuzo II Visual, the archival databases of *Asahi Shimbun* (hereafter, *Asahi*), the newspaper with the second largest circulation. First, we created a database on the subject by extracting articles published between January 1, 2007, and December 31, 2014, and matching the terms “cervical cancer” AND “vaccination” in a keyword search. Then, we tallied the extracted articles based on the number of articles published per month, the number of characters used per month, and the average character count, as well as parsing the articles into the number with a positive or supportive viewpoint, and the number with a negative or oppositional viewpoint. We defined supportive articles as those that included positive comments such as “public funding method of acquisition for programs” or words such as “prevention” and “recommendation.” We defined oppositional articles as those that featured content such as side effects and used terms that shed a negative light on cervical cancer vaccination, such as “refrain from” or “pain.” Additionally, for each positive and negative article, we counted the numbers of articles that included commentary from experts, that gave an explanation about the cervical cancer vaccine, that included information related to consultation, that included photographs or diagrams, and that dealt with compensation. We then conducted a time-series analysis based on this information.

## Results

### Tallied Articles and Basic Characteristics

**Figure 1:**
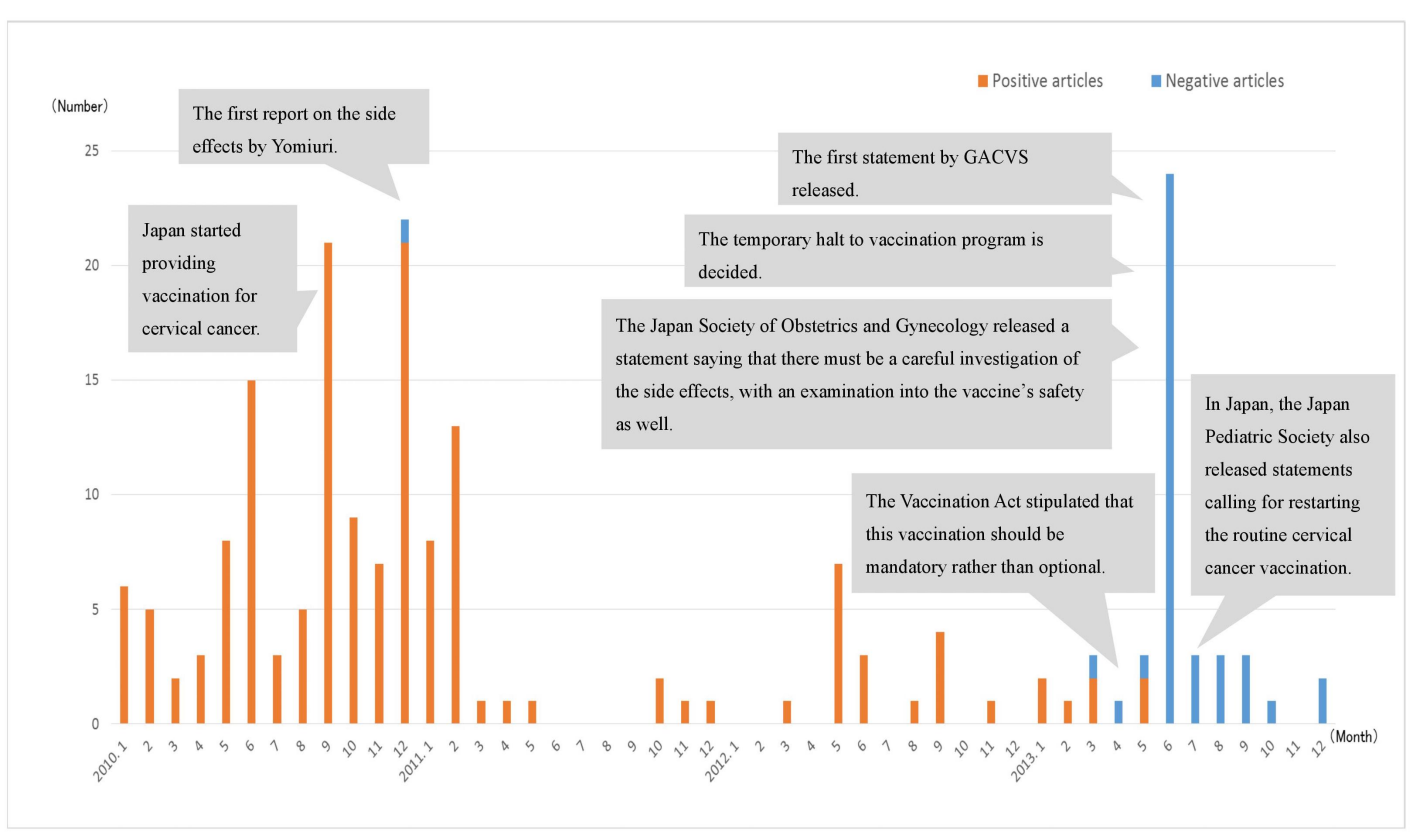
Trends in number of articles related to cervical cancer vaccine (January 2010-December 2013) and their viewpoint (positive or negative). GACVS: Global Advisory Committee on Vaccine Safety.

Figure 1 charts the 219 extracted articles to indicate the change in the number of articles published during the years 2007 to 2014. The first article related to the cervical cancer vaccine was published in *Asahi* on February 4, 2007. The number of articles concerning the cervical cancer vaccine increased during 2010. Overall, there were 154 (70.3%) positive articles and 51 (23.3%) negative articles. Figure 1 further shows the change in the number of positive and negative articles published each month since the start of 2010, when the number of articles being published on the subject increased rapidly, until 2013, when both the regulation of the vaccination program and the articles that specifically covered this issue changed. From September 2010, when a grant was established using public funds for the cervical cancer vaccine, until a temporary interruption in the vaccination program in June 2013, a total of 110 positive articles, which made up 96.5% of all articles up to that point, had been published. However, starting in June 2013, all articles on the subject were negative.

**Table 1:** Tally of articles with positive and negative viewpoints on cervical cancer vaccination and their characteristics (2007-2014).

Characteristics		Year of publication								Total
		2007	2008	2009	2010	2011	2012	2013	2014	
No of articles retrieved		3	0	1	105	31	17	48	14	219
Total no. of characters		3338	0	829	75,354	21,389	21,810	42,198	42,198	207,166
No. of characters per article		1129	0	829	718	690	1283	879	3014	8542
**Positive articles**		2	0	1	97	28	17	7	2	154
	Expert commentary	2	0	1	60	10	6	0	1	80
	Detailed explanation of cervical cancer vaccine	1	0	1	31	8	6	3	1	51
	Places where people could consult experts and obtain advice	1	0	1	10	5	0	0	0	17
	Photographs or charts	0	0	0	13	2	2	0	0	17
	Government compensation	0	0	0	0	1	1	0	0	2
**Negative articles**		0	0	0	1	0	0	39	11	51
	Expert commentary	0	0	0	1	0	0	19	5	25
	Explanation of side effects	0	0	0	1	0	0	29	8	38
	Places where people could consult experts and obtain advice	0	0	0	0	0	0	1	1	2
	Photographs or charts	0	0	0	0	0	0	0	1	1
	Government compensation	0	0	0	0	0	0	3	1	4

### Analysis of the Coverage of and Article Contents on Cervical Cancer Vaccine

A total of 219 articles regarding cervical cancer were published from 2007 through 2014 (Table 1). No articles on the subject were published during 2008, while most (n=105) were published during 2010. During 2014, the highest number of characters per article (n=3014 characters) was allocated to this subject. Of the 154 positive articles, most (n=97) were published in 2010. The content of the positive articles included commentary by knowledge experts (80 articles), which was the most common characteristic of the positive articles, and 60 of the positive articles with expert commentary were published in 2010. Of the positive articles, 51 included a detailed explanation of the cervical cancer vaccine, and most such articles (n=31) were published in 2010. A total of 17 positive articles listed places where people could consult experts and obtain advice, and 10 of these were published in 2010. Also, 17 positive articles included photographs or charts, and most of these (n=13) were published in 2010. Only 2 articles, 1 published in 2011 and 1 in 2012, focused on government compensation.

Of the 51 negative articles, the majority (n=39) were published in 2013. The content of the negative articles had the following characteristics. Of the 25 articles with expert commentary, 19 were published in 2013. A total of 38 articles gave a detailed explanation of side effects, which was the top characteristic of the negative articles, and 29 of these were published in 2013. In total, 2 articles, 1 published in 2013 and 1 in 2014, focused on where people could obtain consultation and advice. Only 1 article, published in 2014, used photographs or charts. Of the 4 articles that mentioned government compensation, 3 were published in 2013.

## Discussion

In this study, we used *Yomiuri* ’s and *Asahi* ’s archival databases to analyze the characteristics of media coverage regarding cervical cancer vaccination in Japan. The first article on cervical cancer vaccination was published by *Asahi* in 2007. There was a marked increase in the number of articles in 2010, and most of them were positive. In 2013, there was an increase in the number of negative articles published.

In 2010, a decision to publicly assist the funding of cervical cancer vaccination led to an increase in the number of articles published that year. On the other hand, the first report on side effects was by *Yomiuri* in a 2010 article titled “Cervical cancer vaccine: many faint, shots on shoulder muscle, severe pain.” This article reported that the research conducted by the Japan Ministry of Health, Labour and Welfare found that one of the side effects of the cervical cancer vaccine was a disruption in balance controlled by the autonomic nervous system, which in many cases causes people to faint [16]. Additionally, the number of articles published on the subject increased in 2013, due to repeated coverage of reported cases about side effects, along with articles about the Ministry of Health, Labour and Welfare recommending discontinuation of the cervical cancer vaccine.

**Figure 2:**
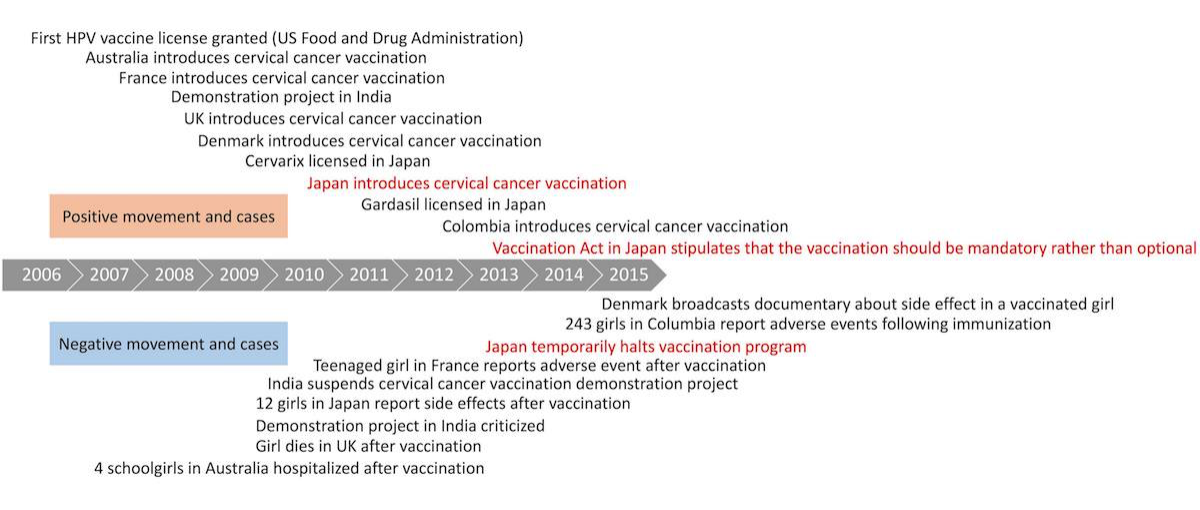
Worldwide trends in cervical cancer vaccination. HPV: human papillomavirus. (Modified by the authors based on Wilson et al [6]).

After June 2013, an advisory body of the World Health Organization, the Global Advisory Committee on Vaccine Safety (GACVS), issued 3 statements to encourage restarting routine cervical cancer vaccination [17,18]. The first statement was made on June 13, 2013. After considering the reports on the side effects of the cervical cancer vaccine in Japan, GACVS reassured readers of the vaccine’s safety. The second statement, made on March 12, 2014, emphasized the effectiveness of the vaccine after giving due consideration to the causal relationship between the cervical cancer vaccine and its side effects. In the third statement, released on December 17, 2015, while referring to the policy decision to temporarily discontinue routine cervical cancer vaccination, the GACVS recommended restarting the vaccination program. In Japan, the Japan Pediatric Society and the Japan Society of Obstetrics and Gynecology also released statements calling for restarting routine cervical cancer vaccination [19,20]. Just 8 days after the government paused routine cervical cancer vaccination, the Japan Society of Obstetrics and Gynecology released a statement calling for a careful investigation of side effects, with an examination into the vaccine’s safety as well. However, our study clarified that the newspaper companies barely covered this. In addition, when we overviewed worldwide trends regarding cervical cancer vaccination (Figure 2), it was clear that there was already information available on side effects, yet no articles mentioned this [6]. Additionally, even though the Japan Medical and Scientific Communications Association was established in 2006, no writers or reporters had knowledge about or covered the subject. This suggests that the Japanese printed news media are out of touch when it comes to medical news reports from outside of Japan.

The research findings indicated that there were very few balanced articles covering the effect and effectiveness of the cervical cancer vaccine, along with its side effects, and most articles were biased and one-sided. Witteman et al reported that biased commentary affects the individual’s health-related activities [21]. With public funds came positive reports, and with the temporary halt in the vaccination program, negative articles appeared. It became obvious that reports by each newspaper relied on official statements by the Ministry of Health, Labour and Welfare and victim organizations. Since the causal relationship between the cervical cancer vaccine and its side effects is yet to be proven scientifically, we suggest that, after reading reports relying on official statements, readers would find it even more difficult to make an accurate decision on this issue. At the same time, Chung noted that parents are far more afraid of losing their children to vaccination than to the disease itself [22]. Some parents have a tendency to not vaccinate their children, and media coverage may be encouraging and influencing such tendencies.

In Japan, every year, approximately 15,000 people are given a diagnosis of cervical cancer, and approximately 3500 die of it [23]. This is the second-highest morbidity rate among female-specific cancers after breast cancer and the highest morbidity rate for cancer among those in their 20s and 30s [24]. As we consider media coverage as a factor affecting human health behavior, the media should extensively report on the cost of not receiving cervical cancer vaccination, global trends concerning cervical cancer vaccination, and statements released by various agencies on the subject.

Since we examined only newspaper articles in this study, to develop this research theme further, television, Internet, and other media coverage beyond printed newspaper articles must be researched as well.

## Abbreviations

GACVS: Global Advisory Committee on Vaccine Safety
HPV: human papillomavirus
