# Figure Caption Correction: Characteristics of Articles About Human Papillomavirus Vaccination in Japanese Newspapers: Time-Series Analysis Study

**DOI:** 10.2196/jmir.9728

**Published:** 2018-03-16

**Authors:** Nao Ueda, Ryoki Yokouchi, Taro Onoda, Atsushi Ogihara

**Affiliations:** ^1^ Graduate School of Human Sciences Waseda University Tokorozawa Japan; ^2^ Department of Health Sciences and Social Welfare School of Human Sciences Waseda University Tokorozawa Japan; ^3^ Department of Medicine School of Medicine Tokai University Isehara Japan; ^4^ Faculty of Human Sciences Waseda University Tokorozawa Japan

The authors of the paper “Characteristics of Articles About Human Papillomavirus Vaccination in Japanese Newspapers: Time-Series Analysis Study” (JMIR Public Health Surveill 2017;3(4):e97) omitted information in the caption of Figure 2. It should have read “Worldwide trends in cervical cancer vaccination. HPV: human papillomavirus. (Modified by the authors based on Wilson et al [6]).”

The corrected article will appear in the online version of the paper on the JMIR website on March 16, 2018, together with the publication of this correction notice. Because this was made after submission to PubMed or Pubmed Central and other full-text repositories, the corrected article also has been re-submitted to those repositories.

Please see the figure with the corrected caption here.

**Figure 2:**
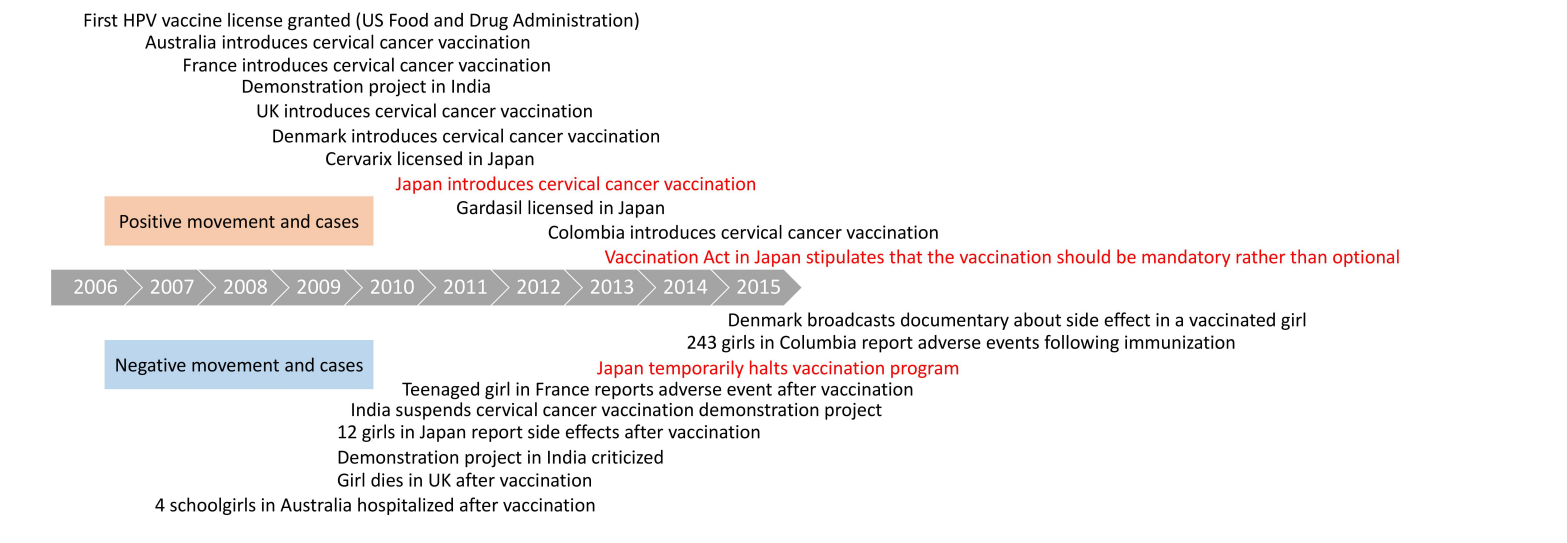
Worldwide trends in cervical cancer vaccination. HPV: human papillomavirus. (Modified by the authors based on Wilson et al [6]).

